# Global Epidemiology of Post-Transplant Lymphoproliferative Disorder (PTLD) in Hematopoietic Stem Cell Transplantation (HSCT): A Systematic Review and Meta-Analysis of Incidence, Subtypes, Risk Factors, and Beyond

**DOI:** 10.3390/jcm15103867

**Published:** 2026-05-18

**Authors:** Shahad Saif Khandker, Alif Hasan Pranto, Afrin Rahman Juthy, Mariam Zaman, Argha Sarkar, Druphadi Sen, Dewan Zubaer Islam, Ehsan Suez, Md Asiful Islam

**Affiliations:** 1 Department of Biochemistry and Molecular Biology, Gono Bishwabidyalay, Dhaka 1344, Bangladesh; shahadsaifkhandker@gmail.com; 2 School of Pharmacy, BRAC University, Dhaka 1212, Bangladesh; alif.hasan.pranto@g.bracu.ac.bd (A.H.P.); moriombracu@gmail.com (M.Z.); arghaasarkar820@gmail.com (A.S.); 3 Faculty of Engineering and Science, University of Greenwich, Central Avenue, Chatham ME4 4TB, UK; afrinrahman85@gmail.com; 4 Department of Cell and Systems Biology, University of Toronto, 27 King’s College Cir, Toronto, ON M5S 1A1, Canada; sendruphadi@gmail.com; 5 Department of Microbiology, Jahangirnagar University, Dhaka 1342, Bangladesh; dewanzubaerislam@gmail.com; 6 Institute of Bioinformatics, University of Georgia, Athens, GA 30602, USA; ehsan.suez@uga.edu; 7 Department of Biomedical Science and Physiology, School of Pharmacy and Life Sciences, Faculty of Science and Engineering, University of Wolverhampton, Wolverhampton WV1 1LY, UK

**Keywords:** PTLD, HSCT, transplant, malignant, frequency, incident

## Abstract

**Background:** Hematopoietic stem cell transplantation (HSCT) is a procedure used in different malignant and non-malignant diseases. Although post-transplant lymphoproliferative disorder (PTLD) is infrequently observed in patients with HSCT, no study on the overall global incidence rate is available to date. **Methodology:** In this study, we selected 39 studies from 941 studies from three databases (i.e., PubMed, ScienceDirect, and Google Scholar) to identify the global incidence rate of PTLD in HSCT. **Results:** The pooled incidence was determined to be 5.6% (95% CI: 5.0 to 6.3) and rose further to 12.4% (95% CI: 10.2 to 14.7) after excluding outlier studies. The quality of the studies was high as well. PTLD was prevalent the most in allogenic HSCT (i.e., 5.6% (95% CI: 4.9 to 6.3)) and within the European region (i.e., 27.1% (95% CI: 21.4 to 32.8)). Among risk factors, human leukocyte antigen (HLA) mismatch was reported in most of the studies. **Conclusions:** This study assessed and discussed the overall global incidence of PTLD in HSCT patients, continent-based incidence, and risk factors that can be helpful in finding the possible prevention mechanism of PTLD and implementing individualized treatment approaches based on the treatment availability during HSCT.

## 1. Introduction

Transplantation is a life-saving medical procedure involving grafting cells, tissues, or organs to replace or repair damaged ones, thereby enhancing the quality of life in cases of organ failure [1]. Hematopoietic stem cell transplantation (HSCT), a subset of transplantation therapies, exemplifies this potential, offering hope for patients with malignant and non-malignant diseases [2]. Stem cells, particularly hematopoietic stem cells (HSCs), play a crucial role in reconstructing the immune system, which also helps to foster immune tolerance and improve post-transplant outcomes [3]. While advancements in transplant techniques have significantly increased survival rates, they also come with risks. Among the most serious complications is post-transplant lymphoproliferative disorder (PTLD) [4].

PTLD refers to a heterogeneous group of lymphoid proliferative conditions ranging from benign, self-limiting infectious mononucleosis to aggressive lymphomas [5]. Usually, after transplantation, patients who experience fever, adenopathy, weight loss, mass lesions, indescribable pain, or dysfunction of the transplanted organ can be recognized as a suspect for PTLD [6]. Although PTLD in the solid-organ transplant population initiates after 6 months and in HSCT recipients after 70–90 days, some reports claim that the onset of PTLD can occur even within 1 week or even after 9 years after the transplantation. Symptoms are diverse and may be related to organ dysfunction, mass effect, viremia, or lymphoma-related B symptoms. The reasons for these differences in incidence were not clearly reported; however, depending on the amount of lymphoid tissue present in each organ, immunological reaction, virus, and immunosuppression treatment, it may plausibly vary [6,7].

Despite studies identifying PTLD in various solid-organ transplant recipients, the association and incidence of PTLD in HSCT patients remain unclear. Therefore, in this systematic review and meta-analysis, we determined the incidence of PTLD in HSCT patients along with subgroup analyses of the incidence of PTLD among different types of HSC transplantation (i.e., allogeneic, autologous, and syngeneic), continent-based analyses of PTLD incidence in HSCT, and the possible risk factors.

## 2. Methodology

### 2.1. Study Design and Selection of Inclusion–Exclusion Criteria

This study followed the Preferred Reporting Items for Systematic Reviews and Meta-Analyses 2020 (PRISMA 2020) study design with the PRISMA checklist 2020 (Supplementary File S1) (registration links: PROSPERO 2026 CRD420261372894 (https://www.crd.york.ac.uk/PROSPERO/view/CRD420261372894 (accessed on 5 April 2026)) and OSF: https://osf.io/mp72n (accessed on 5 April 2026)), with minor adjustments, and employed a systematic approach to identify relevant studies and conduct a meta-analysis to assess the global incidence of PTLD among HSCT patients, adhering to established PRISMA guidelines obtained from previous studies [8,9]. The study included peer-reviewed articles focusing on the incidence, prevalence, occurrence, or frequency of PTLD in HSCT patients. Excluded materials encompassed narrative or systematic reviews, meta-analyses, book chapters, case reports, editorials, correspondence, conference papers, press releases, and non-original journal articles. Only English-language publications were considered. The study did not involve patients, the public, healthcare institutions, or any third parties in data collection, processing, study design, assessment, analysis, or interpretation of results.

### 2.2. Search Strategy and Study Inclusion

A comprehensive search strategy was developed to screen relevant studies from three online databases: PubMed, ScienceDirect, and Google Scholar. The search utilized multiple terms such as “prevalence,” “post-transplant lymphoproliferative disorder,” and “hematopoietic stem,” along with their respective related terms or short forms like “prevalence”, “epidemiology”, “frequency”, “PTLD”, “bone marrow”, and “transplant”. Boolean operators (AND/OR) were used to refine the search. Specific filters, including “Title/Abstract” for PubMed, “Title, abstract or author-specified keywords” for ScienceDirect, and “allintitle” for Google Scholar, were applied in the advanced search options, following previous studies with slight modifications [10]. No publication date filters were used. Duplicate search results were carefully managed and excluded from the study.

### 2.3. Data Extraction, Meta-Analysis, and Heterogeneity

The authors independently conducted the initial search and necessary data extraction from the included studies, subsequently validating their findings through discussion. Primarily, the data of PTLD cases and the number of HSCT patients were obtained from each of the individual studies. The extracted information encompassed basic study characteristics such as study ID, study type, number of HSCT participants, male and female numbers, age, HSCT type, PTLD type, and study region. Any disagreements regarding data inclusion or exclusion were carefully resolved by the authors, and they addressed missing or unclear information by contacting the corresponding or first author of the study.

The authors opted for a meta-analysis approach to determine the pooled incidence of PTLD in HSCT patients based on the collected data. They employed a random-effects model with a 95% confidence interval (CI) to analyze the pooled incidence. The I^2^ statistics method was used to estimate the pooled heterogeneity of the included studies. The I^2^ values indicated the level of heterogeneity among the studies, with 25–50% suggesting low heterogeneity, 51–75% indicating moderate heterogeneity, and values exceeding 75% signifying high heterogeneity. Additionally, outlier studies and sensitivity analysis were implemented, based on previously established methodologies, to explore potential outliers and their influence on the overall effect size [11,12]. The outlier determination and sensitivity analyses were performed sequentially: first, the primary exclusion of outlier studies was identified using funnel plots, along with Egger’s and Begg’s tests, followed by the further assessment using Galbraith plots and, finally, the reanalysis of the primary meta-plot using a random-effects model. The meta-analysis, forest plot, funnel plot, and Galbraith plot construction were performed using RStudio software (Boston, MA, USA, version 4.3.0) and the “metafor” package (version 4.2-0) of R.

### 2.4. Evaluation of Study Quality and Analysis of Bias Risk

The researchers evaluated the quality of the included studies using a set of nine questions derived from the Study Quality Assessment Tools, National Institutes of Health (NIH), and Systematic Reviews: Step 6: Assess Quality of Included Studies, University of North Carolina (UNC) [13,14]. In this study, the selected questions could be answered with “Yes”, “No”, “Unclear”, “Not Reported” (NR), or “Not Applicable” (NA), which were then converted to numerical scores (e.g., 1 for Yes, 0 for No and Unclear, and no score for NR and NA). For each study, these scores were totaled and divided by the number of questions (9 in this case), then converted to a percentage. The resulting percentage indicated the study’s quality and potential publication bias risk: <50% suggested low quality and high bias risk, 50–70% indicated moderate quality, 70–80% suggested moderately high quality, and >80% signified high quality and low bias risk. The scoring method was adapted from previous research with minor modifications [15,16].

### 2.5. Subgroup Analyses and Risk Factors

To evaluate the PTLD incidence among HSCT patients based on the type of PTLD, continent-based subgroup analyses were conducted. Plausible risk factors of the PTLD were also assessed.

## 3. Results

### 3.1. Search Results and Study Inclusion

**Figure 1:**
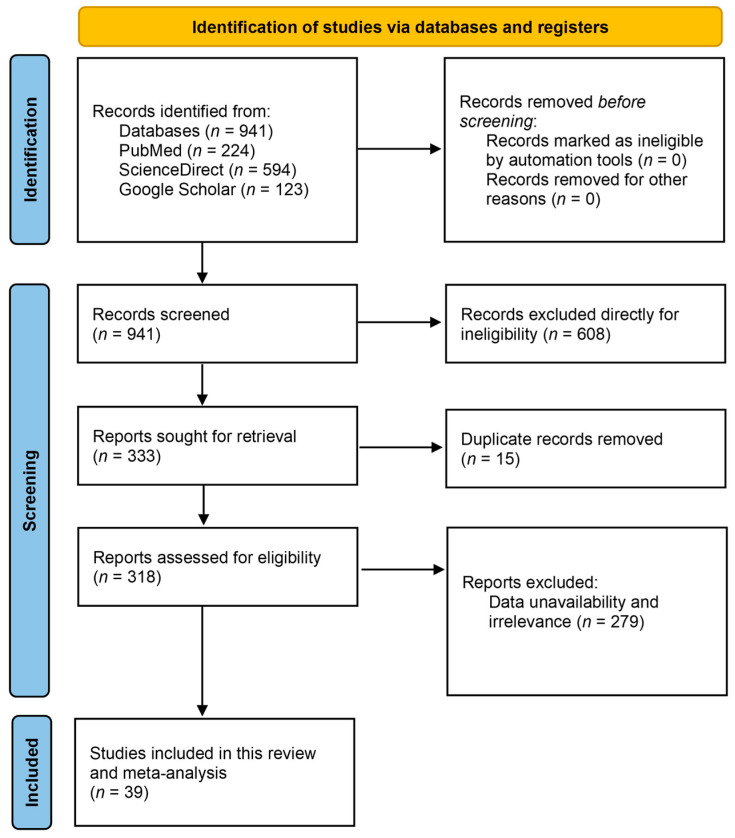
A simplified PRISMA flow diagram of the methodology.

Initially, a total of 941 search results were identified from three electronic databases, PubMed (*n* = 224), ScienceDirect (*n* = 594), and Google Scholar (*n* = 123), based on our search strategies and applied keywords, as well as various filters used in different databases during searches. In the study screening step, 608 articles were directly excluded, considering they included reviews, systematic reviews, mini-reviews, meta-analyses, editorials, case reports, and correspondence other than full-text original research articles. In total, 333 studies proceeded to the eligibility assessment step, from which 15 studies were excluded due to study duplication. Then, 50 articles were selected for evaluation, and after rigorous screening, validation, and excluding studies (*n* = 279) that were deemed to be irrelevant to our study aims and objectives, 39 studies were selected to be included in our systematic review and meta-analysis as they matched our eligibility and inclusion criteria properly (Figure 1).

### 3.2. Major Characteristics of the Included Studies

The authors carefully extracted and validated major characteristic data of the included articles (*n* = 39) that completely met the criteria of the current systematic review and meta-analysis [17,18,19,20,21,22,23,24,25,26,27,28,29,30,31,32,33,34,35,36,37,38,39,40,41,42,43,44,45,46,47,48,49,50,51,52,53,54,55]. The 39 included studies were conducted in different countries of different continents, including Europe (i.e., Portugal, Italy, Germany, Switzerland, Sweden, Spain, France, Poland, and Denmark), Asia (i.e., India, Japan, China, Korea, and Turkey), and North America (i.e., Canada and the USA). Among HSCT types, allogenic, autologous, and syngeneic types were reported. Among the types of PTLD, Monomorphic PTLD (M-PTLD), Polymorphic-PTLD (P-PTLD), plasmacytic hyperplasia PTLD (PH-PTLD), classic Hodgkin lymphoma PTLD (CHL-PTLD), Epstein–Barr virus PTLD (EBV-PTLD), and early lesion PTLD (EL-PTLD) were reported. In addition, the type of study was mainly cross-sectional or cohort. The numbers of total participants, along with male and female numbers and their age details, are reported in detail in Table 1.

### 3.3. Primary Meta-Analysis, Heterogeneity, Outliers and Sensitivity

**Figure 2:**
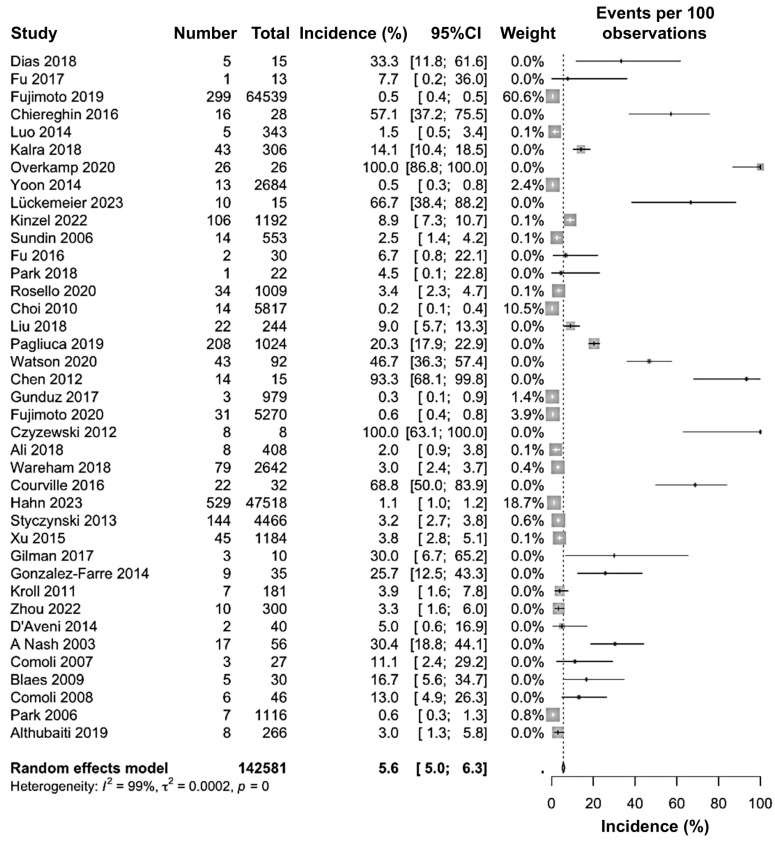
Forest plot of the pooled incidence of PTLD in patients after hematopoietic stem cell transplantation [17,18,19,20,21,22,23,24,25,26,27,28,29,30,31,32,33,34,35,36,37,38,39,40,41,42,43,44,45,46,47,48,49,50,51,52,53,54,55].

The primary objective of our meta-analysis was to determine the pooled incidence of PTLD in HSCT patients globally. As a result, 1822 PTLD cases were reported out of 142,581 HSCT patients. Based on data extracted from the included studies, an overall pooled incidence of 5.6% (95% CI: 5.0 to 6.3) with a substantial heterogeneity of 99% (*p* = 0) was found. The highest incidence was found in Overkamp 2020 (100%, (95% CI: 86.8 to 100.0)) [23] and Czyzewski 2012 (100%, (95% CI: 63.1 to 100.0)) [38], whereas the lowest incidence was found in Choi 2010 (0.2% (95% CI: 0.1 to 0.4)) [31]. The weight of some studies was calculated as 0.0% due to the lower sample size compared to the studies that had a larger sample size; however, that did not have any correlation with the pooled incident value (Figure 2). 

**Figure 3:**
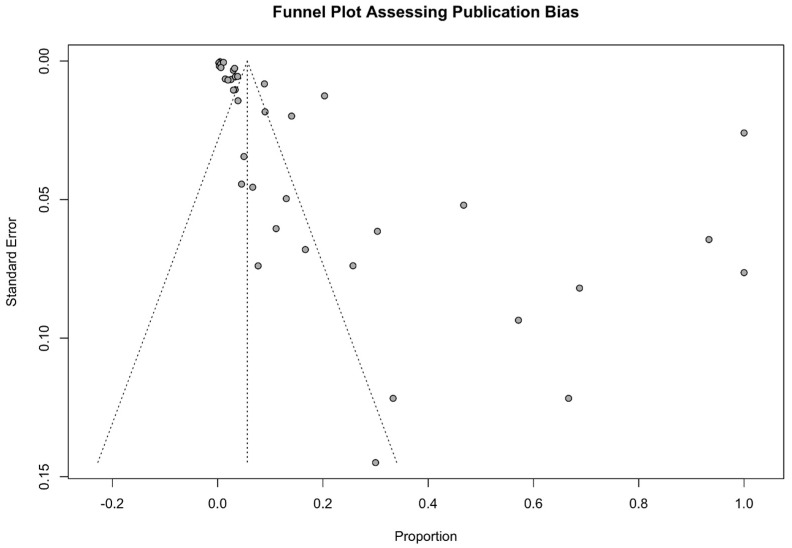
Funnel plot assessing the plausible publication biases of included studies for the incidence of PTLD in patients after hematopoietic stem cell transplantation. Here, the value of Egger’s test was identified as z = 6.95, *p* < 0.0001 and the value of Begg’s test was identified as Kendall’s tau = −0.32, *p* = 0.0058, where both tests indicated asymmetry.

A funnel plot constructed for visualization of the possible sources of publication bias indicated multiple potential outlier studies with high risks of bias. The funnel plot asymmetry was identified by Egger’s test (z = 6.95, *p* < 0.0001) and Begg’s test (Kendall’s tau = −0.32, *p* = 0.0058), where both tests indicated asymmetry (Figure 3).

**Figure 4:**
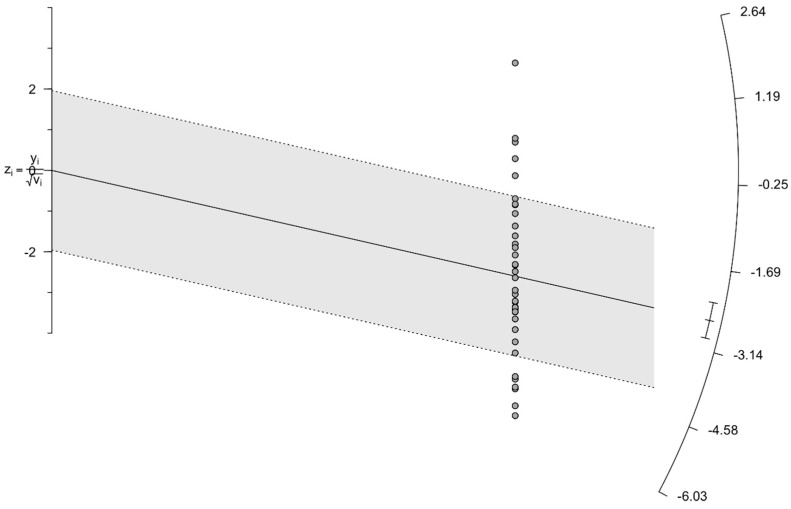
Galbraith’s plot assessing the outlier studies for the incidence of PTLD in patients after hematopoietic stem cell transplantation.

The Galbraith plot, which was constructed for visual investigation and confirmation of the potential sources of bias indicated 11 outlier studies (i.e., Chen 2012 [35], Courville 2016 [41], Luckemeier 2023 [25], Chiereghin 2016 [20], Watson 2020 [34], Park 2006 [54], Fujimoto 2020 [37], Yoon 2014 [24], Fujimoto 2019 [19], Gunduz 2017 [36], and Choi 2010 [31]), as shown in Figure 4.

**Figure 5:**
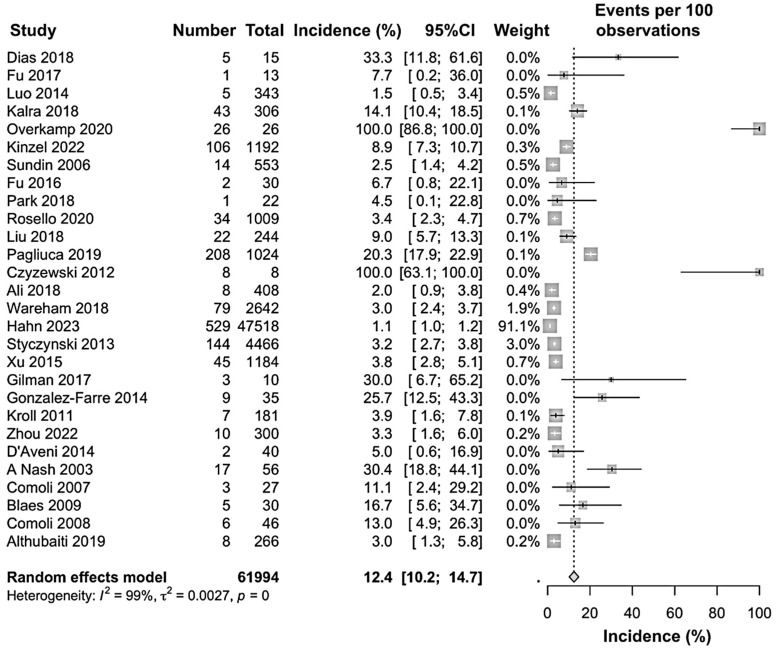
Forest plot excluding the outlier studies of the incidence of PTLD in patients after hematopoietic stem cell transplantation [17,18,21,22,23,26,27,28,29,30,32,33,38,39,40,42,43,44,45,46,47,48,49,50,51,52,53,55].

After excluding the outliers, the forest plot was reconstructed using a random effect model to identify the sensitivity. As a result, the incidence was enhanced to 12.4% (95% CI: 10.2 to 14.7) as compared to the primary outcome; however, the heterogeneity remained high (I^2^ = 99%) (Figure 5).

### 3.4. Assessment of Study Quality and Risk of Bias

Based on the answers to our specified set of quality assessment questions and subsequent scoring strategy, 33 studies scored more than 80%, indicating high-quality studies, and 6 studies scored more than 50%, resembling moderate-quality studies, among which 3 studies have scored above 70%, resembling moderately high-quality studies. Interestingly, 17 studies obtained a 100% score. No study was determined to be scored less than 50%; hence, there were no low-quality studies with a high risk of bias (Table 2).

### 3.5. Subgroup Analysis and Risk Factors

**Figure 6:**
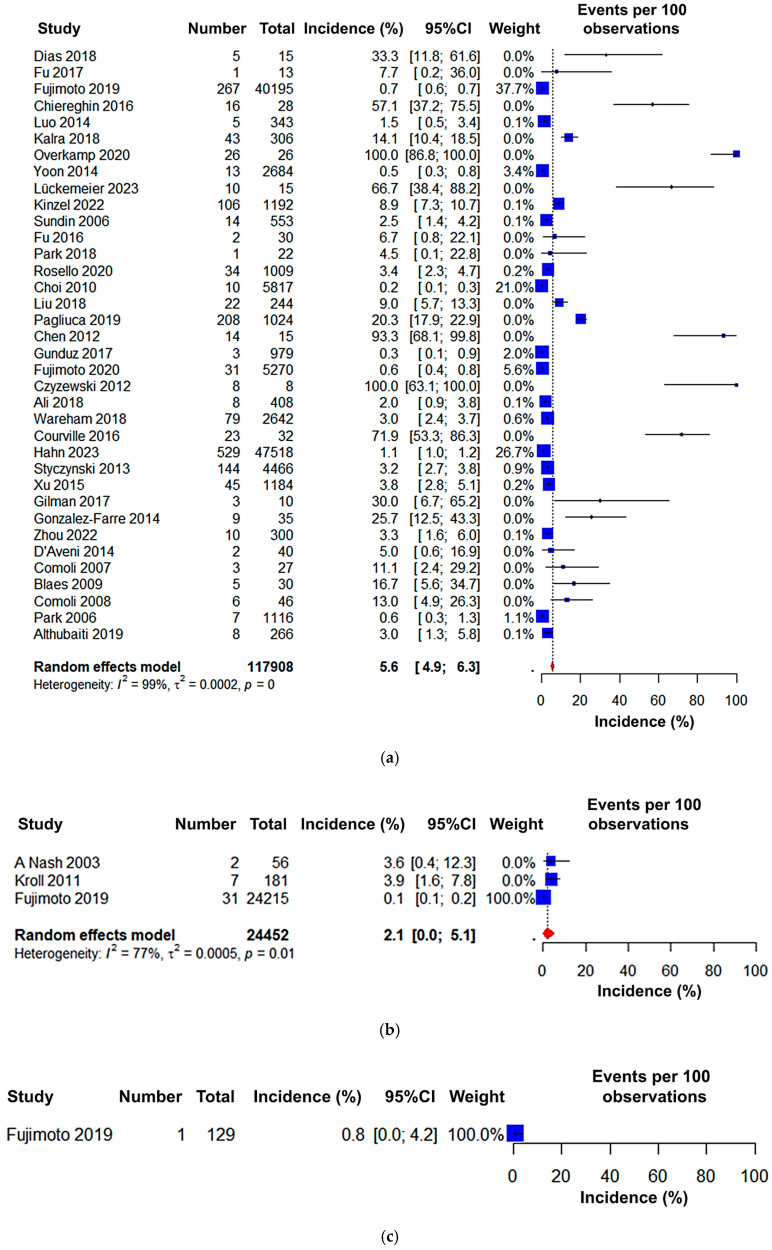
Forest plot of the incidence of PTLD in patients after (**a**) allogeneic [17,18,19,20,21,22,23,24,25,26,27,28,29,30,31,32,33,35,36,37,38,39,40,41,42,43,44,45,46,48,49,51,52,53,54,55], (**b**) autologous [19,47,50], and (**c**) syngeneic hematopoietic stem cell transplantation [19].

Subgroup analysis was aimed at investigating the pooled incidence of PTLD in HSCT patients based on the type of HSCT and patients across different continents. For HSCT type, the highest pooled incidence was found in allogeneic HSCT (5.6% (95% CI: 4.9 to 6.3)) followed by the autologous (2.1% (95% CI: 0.0 to 5.1)) and syngeneic (0.8% (95% CI: 0.0 to 4.2)) HSCT (Figure 6).

**Figure 7:**
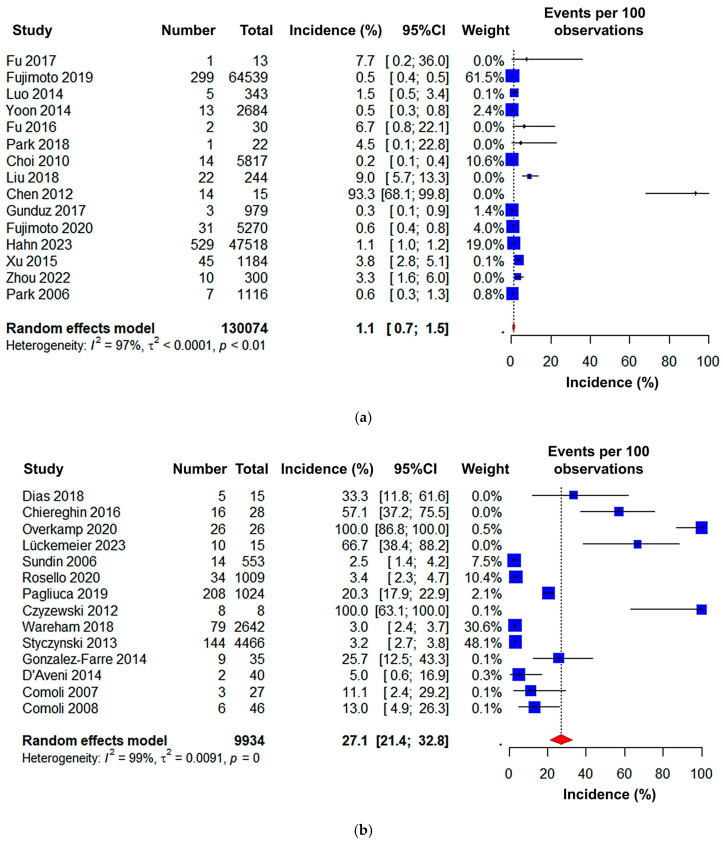

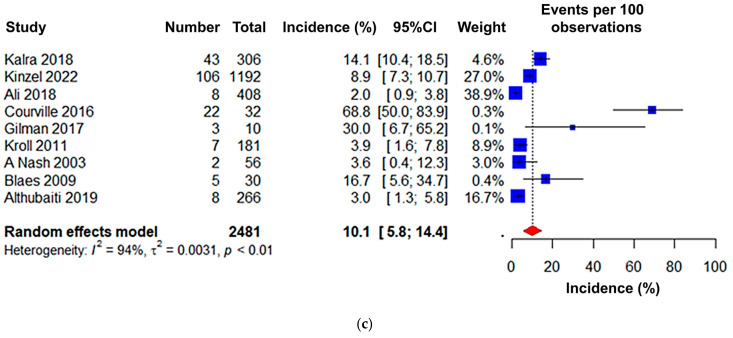
Forest plot of the pooled incidence of PTLD in patients after HSCT in (**a**) Asia [18,19,21,24,28,29,31,32,35,36,37,42,44,48,54], (**b**) Europe [17,20,23,25,27,30,33,38,40,43,46,49,51,53], and (**c**) North America [22,26,39,41,45,47,50,52,55].

**Figure 8:**
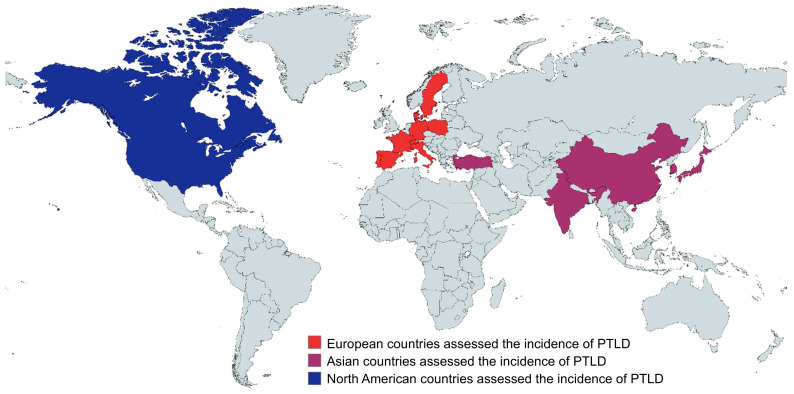
Geographical locations of the studies reporting the incidence of PTLD in HSCT patients.

Regarding the continent-based subgroup analysis, Asia was found to have the lowest incidence rate (1.1% (95% CI: 0.7 to 1.5)), followed by North America (10.1% (95% CI: 5.8 to 14.4)), whereas Europe had the highest incidence rate of PTLD among HSCT patients (27.1% (95% CI: 21.4 to 32.8)) (Figure 7). The geographical locations of the countries within different continents are demonstrated in detail in Figure 8.

**Figure 9:**
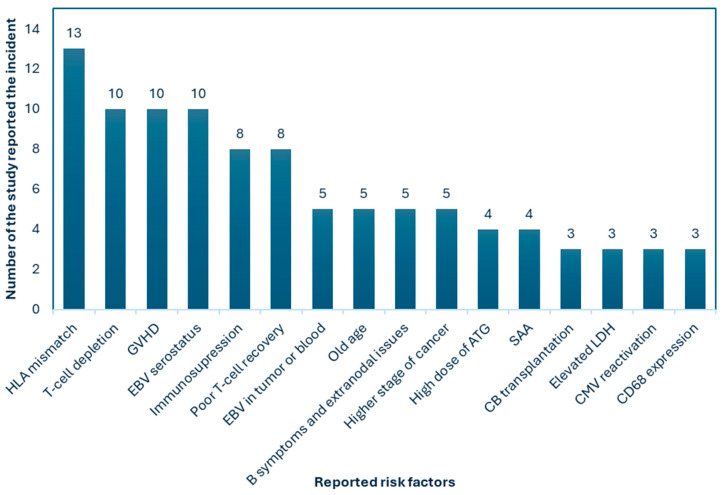
Plausible risk factors of PTLD reported in the different included studies. Here, GVHD: graft-versus-host disease; EBV: Epstein–Barr virus; ATG: Antithymocyte Globulin; SAA: severe aplastic anemia; CB: cord blood; LDH: lactate dehydrogenase; CMV: cytomegalovirus.

Plausible reported risk factors were also obtained from the included studies. As a result, we observed that HLA mismatch was the highest reported risk factor of PTLD, including all types of HSCT, followed by T-cell depletion, graft-versus-host disease, and EBV seroincidence or serostatus. All the plausible risk factors are presented in Figure 9.

## 4. Discussion

To the best of the authors’ knowledge, this is the first meta-analysis to investigate the global incidence of PTLD among patients undergoing HSCT. By analyzing data from diverse regions, we provide new insights into the incidence of PTLD with HSCT, highlighting significant epidemiological and clinical trends. Our findings determined that the pooled incidence rate of PTLD among HSCT was 5.6% (95% CI: 5.0 to 6.3), which indicates that it is a critical concern for HSCT patients, and they need to be carefully handled after the transplantation to restrict PTLD, as the incidence rate is quite high (Figure 2). Interestingly, after excluding the plausible outlier studies, the incidence rate of PTLD rose to 12.4% (95% CI: 10.2 to 14.7), which further proves it is an even more serious issue for HSCT patients (Figure 5). According to previous reports, the incidence of PTLD varies depending on the type of transplantation, such as 19% for intestinal transplants, 2–10% for heart transplants, 5–9% for heart–lung transplants, and 2–8% for liver transplants [7]. Therefore, other than PTLD in intestinal transplant patients, every other reported transplantation was investigated to have a lower incidence of PTLD.

In subgroup analyses, we initially investigated that allogenic HSCT was the most prevalent transplantation type as compared to autologous and syngeneic HSCT. However, the incidence of PTLD was also higher in the allogenic HSCT (5.6% (95% CI: 4.9 to 6.3) as compared to autologous (2.1% (95% CI: 0.0 to 5.1) and syngeneic (0.8% (95% CI: 0.0 to 4.2) HSCT (Figure 6). These differences in PTLD among autologous, syngeneic, and allogeneic HSCT highlight the need for stratified transplant approaches. Autologous HSCT, which uses the patient’s own stem cells, eliminates concerns about donor compatibility and reduces the risk of graft-versus-host disease (GVHD). In addition, in the autologous transplantation process, immune reconstitution occurs more rapidly than following an allogeneic transplant, and there is a reduced risk of opportunistic infections [56,57]. Again, in autologous transplant, immunosuppression is not required for graft rejection, as it is required for allogenic transplantation; therefore, it is a more promising process [58]. Our findings indicate that autologous HSCT recipients have substantially lower rates of PTLD, with near-zero post-transplant mortality (Figure 6). Syngeneic HSCT, on the other hand, performed between identical twins, shows promise but remains underexplored, with limited data available on its efficacy and long-term outcomes [59,60]. Interestingly, patients who develop PTLD may have other risk factors that can create critical conditions leading to death. Physiological conditions such as age, weakness, comorbidities such as diabetes, cardiovascular diseases, and psychological complications such as anxiety and depression were some factors that may lead to death [61,62,63].

Depending on the study region, we did a separate subgroup analysis, observing that the studies were from three different continents: Asia, Europe, and North America. Interestingly, we found that the Asian region had the lowest PTLD rate (1.1% (95% CI: 0.7 to 1.5)) compared to the European (27.1% (95% CI: 21.4 to 32.8)) and North American (10.1% (95% CI: 5.8 to 14.4)) regions, respectively (Figure 8 and Figure 9). Plausible risk factors or other physiological conditions and comorbidities may impact these changes in the incidence rate. In addition, we did not find or could not include any autologous HSCT-based study performed in Europe, which may also have had an impact on its higher incidence rate. Additionally, EBV is acquired at younger ages in Asia than in Europe and North America, with Asian seroprevalence exceeding 80% by age 5, meaning most Asian transplant recipients are already EBV-seropositive before transplantation [64]. This minimizes the high-risk donor-positive/recipient-negative (D+/R−) mismatch, the most important risk factor for PTLD, which is substantially more prevalent among Western recipients [65]. Moreover, T-cell depletion, a major risk factor for EBV reactivation, and more aggressive T-cell--depleting regimens, more commonly reported in European studies, markedly amplify PTLD susceptibility [66].

On the other hand, among the overall HSCT risk factors we determined in our included articles, HLA mismatch was the most prevalent, followed by T-cell depletion, GVHD, and EBV infection. In addition, immunosuppression, poor T-cell recovery, presence of EBV in tumor/blood, old age, B symptoms (i.e., sweats, pyrexia, and weight loss), and extranodal involvements (i.e., lung, bone marrow, gastrointestinal tract (GIT), skin, and central nervous system (CNS)), presence of high stage of cancer, high dose of Antithymocyte Globulin (ATG), severe aplastic anemia (SAA), cord blood (CB) transplantation, elevated level of lactate dehydrogenase (LDH), cytomegalovirus (CMV) reactivation, and CD68 expression are some other reported risk factors. Previous studies also suggest these risk factors are crucial [67,68]. It is also reported that the development of PTLD is closely linked to immunosuppression, particularly the use of ATG, ex vivo T-cell depletion, and mismatched or unrelated donors [69]. These factors weaken the immune system’s ability to control EBV-driven B-cell proliferation, significantly increasing the risk of PTLD. Umbilical cord blood grafts further elevate PTLD risk due to their lower T-cell maturity and count [70]. Advances in transplant techniques, such as improved T-cell depletion protocols, have reduced traditional complications but introduced new challenges, including EBV-related PTLD [71]. EBV infection was found to be prevalent among 90% of the young population worldwide. Usually, it remains in a latent and asymptomatic state waiting for reactivation while the immune system is weakened [72]. Therefore, although solid-organ transplantation (SOT) and HSCT differ in the transplanted organ type and process, EBV reactivation can occur in both cases. However, in HSCT, particularly in haploidentical HSCT or allogenic HSCT with a risk of GVHD, where immunosuppression is applied, EBV reactivation should be carefully monitored, whereas in SOT, commonly, the use of immunosuppressive drugs increases the risk of EBV reactivation [73,74]. A reduction in immunosuppression (RI), a strategy that decreases immunosuppressive drug doses to enhance endogenous anti-EBV immunity without fully discontinuing therapy, remains the first-line treatment for PTLD, aiming to restore cytotoxic T lymphocyte (CTL) function while minimizing allograft rejection [71]. For cases with CD20-positive PTLD, rituximab and anti-CD20 monoclonal antibodies have shown promising results, achieving remission rates of 40–60% when combined with chemotherapy.

The heterogeneity was determined to be high in this study. The high heterogeneity usually results from multiple factors, including study types, participant types, sample sizes and types, treatment variations, etc. [12,75]. Previously, prevalence-based studies also found higher heterogeneity for similar reasons [9,76]. Since, in this study, the number of participants, study types, and treatment outcomes varied widely, the heterogeneity may have increased.

The increasing incidence of PTLD in recent decades highlights the evolving landscape of transplantation. Novel therapies, including chimeric antigen receptor (CAR) T cells targeting CD19 and EBV-specific adoptive T-cell therapies, are being explored as potential transformative approaches in PTLD management. Factors such as expanded use of HSCT, potent immunosuppressive drugs, and improved diagnostic techniques have contributed to this trend. However, these advances also present opportunities for early detection and intervention, which are critical for improving survival rates [67,77,78,79]. Nevertheless, routine checks and follow-ups are crucial for diagnosing and managing PTLD, enabling clinicians to initiate preemptive therapies before the onset of clinical symptoms.

## 5. Limitations of This Study

We could not include data from general search engines, news portals, blogs, or other databases. Data from only published articles were included. In addition, from the final search to the completion of the total work, a few more research articles may have been published, which we could not include. We also acknowledge that the pooled estimate may convey low certainty because the sources of heterogeneity are not systematically explored. In addition, in subgroup analyses, especially in autologous and syngeneic HSCT, we found a lower number of studies and data, which is another limitation of this study. Again, we could not separate the risk factors based on the type of HSCT.

## 6. Conclusions

This study represents a crucial step forward in understanding the incidence and connection of PTLD in HSCT patients. Our findings emphasize the need for early diagnosis, personalized treatment strategies, and continued research into the mechanisms underlying PTLD. The high heterogeneity might hinder the exact outcome and indicate the necessity of further research. However, by addressing these challenges, positive outcomes for HSCT recipients and the advancement of the field of transplantation medicine can be achieved.

## Figures and Tables

**Table 1:** General characteristics of the included studies.

Study ID	Study Type	HSCT Participants	Male	Female	Patient Age (Y) (Median Range)	HSCT Types	PTLD Types	Region	Reference
Dias 2018	Cross-sectional study	15	7	8	NR	Allogeneic	M-PTLD, P-PTLD	Portugal	[17]
Fu 2017	Cross-sectional study	13	9	4	NR (18–46)	Allogeneic	M-PTLD	India	[18]
Fujimoto 2019	Cohort study	64,539	NR	NR	NR	Autologous, Allogeneic, Syngeneic	NR	Japan	[19]
Chiereghin 2016	Cohort study	28	20	8	NR	Allogeneic	EBV-PTLD	Italy	[20]
Luo 2014	Cross-sectional study	343	260	83	NR	Allogeneic	NR	China	[21]
Kalra 2018	Cross-sectional study	306	NR	NR	NR	Allogeneic	NR	Canada	[22]
Overkamp 2020	Cohort study	26	NR	NR	NR	Allogeneic	M-PTLD, P-PTLD, CHL-PTLD,	Germany	[23]
Yoon 2014	Cohort study	2684	NR	NR	26 (NR)	Allogeneic	M-PTLD, P-PTLD, PH-PTLD	South Korea	[24]
Lückemeier 2023	Cohort study	15	11	4	NR (32–67)	Allogeneic	M-PTLD, P-PTLD, CHL-PTLD	Switzerland	[25]
Kinzel 2022	Cohort study	1192	674	518	45 (NR)	Allogeneic	M-PTLD, P-PTLD,	Canada	[26]
Sundin 2006	Cohort study	553	NR	NR	NR	Allogeneic	EBV-PTLD	Sweden	[27]
Fu 2016	Cross-sectional study	30	19	11	23 (14–52)	Allogeneic	M-PTLD, P-PTLD	China	[28]
Park 2018	Cross-sectional study	22	11	11	9 (1.6–16.9)	Allogeneic	EBV-PTLD	Korea	[29]
Rosello 2021	Cohort study	1009	597	412	41 (14–70)	Allogeneic	M-PTLD, P-PTLD	Spain	[30]
Choi 2010	Cross-sectional study	5817	NR	NR	42.6 (24–60)	Allogeneic	M-PTLD, P-PTLD	Korea	[31]
Liu 2018	Cross-sectional study	244	NR	NR	NR	Allogeneic	NR	China	[32]
Pagliuca 2019	Cross-sectional study	1024	NR	NR	42.5 (8.3–74.7)	Allogeneic	EBV-PTLD, M-PTLD, P-PTLD	France	[33]
Watson 2020	Cohort study	92	NR	NR	47.1 (17–75)	Autologous, Allogeneic	NR	USA	[34]
Chen 2012	Cross-sectional study	15	12	3	31 (9–60)	Allogeneic	M-PTLD, P-PTLD, PH-PTLD, EL-PTLD	China	[35]
Gunduz 2017	Cohort study	979	NR	NR	33 (5–71)	Allogeneic	CHL-PTLD	Turkey	[36]
Fujimoto 2020	Cohort study	5270	3193	2077	47 (16–88)	Allogeneic	NR	Japan	[37]
Czyzewski 2012	Cross-sectional study	8	NR	NR	16 (5–19)	Allogeneic	EBV- PTLD, M-PTLD, P-PTLD	Poland	[38]
Ali 2018	Cross-sectional study	408	NR	NR	5.9 (2.3–17.3)	Allogeneic	EBV- PTLD, M-PTLD, P-PTLD	Canada	[39]
Wareham 2018	Cohort study	2642	1581	1061	42.9 (17–35)	Allogeneic	EBV-PTLD	Denmark	[40]
Courville 2016	Cross-sectional study	32	19	13	44 (3–72)	Allogeneic	M-PTLD, P-PTLD	USA	[41]
Hahn 2023	Cohort study	47,518	29,528	17,990	5.32 (10–60)	Allogeneic	NR	Korea	[42]
Styczynski 2013	Cross-sectional study	4466	NR	NR	30 (10–68)	Allogeneic	EBV- PTLD	Poland	[43]
Xu 2015	Cohort study	1184	NR	NR	27 (3–49)	Allogeneic	NR	China	[44]
Gilman 2017	Cross-sectional study	10	NR	NR	47 (19–71)	Allogeneic	EBV- PTLD, M-PTLD, P-PTLD, PH-PTLD	Netherlands	[45]
Gonzalez-Farre 2014	Cross-sectional study	35	26	9	54 (26–77)	Allogeneic	P-PTLD, PH-PTLD, CHL-PTLD	Spain	[46]
Kroll 2011	Cross-sectional study	181	NA	NA	55 (37–73)	Autologous	EBV-PTLD	USA	[47]
Zhou 2022	Cohort study	300	184	116	28 (NR)	Allogeneic	EBV-PTLD	China	[48]
D’Aveni 2014	Cohort study	40	20	20	30 (NR)	Allogeneic	EBV-PTLD	France	[49]
A Nash 2003	Cross-sectional study	56	21	35	42 (NR)	Autologous	EBV-PTLD	USA	[50]
Comoli 2007	Cross-sectional study	27	15	12	8 (NR)	Allogeneic	EBV-PTLD	Italy	[51]
Blaes 2009	Cohort study	30	NR	NR	55 (NR)	Allogeneic	EBV-PTLD	USA	[52]
Comoli 2007	Cross-sectional study	46	NR	NR	NR	Allogeneic	EBV-PTLD	Italy	[53]
Park 2006	Cohort study	1116	NR	NR	NR (17–45)	Allogeneic	EBV-PTLD	Korea	[54]
Althubaiti 2019	Cohort study	266	NR	NR	NR	Allogeneic	EBV-PTLD	Canada	[55]

NR: not reported; Y: years; PTLD: post-transplant lymphoproliferative disorder; HSCT: hematopoietic stem cell transplantation; M-PTLD: Monomorphic PTLD; P-PTLD: Polymorphic PTLD; PH-PTLD: plasmacytic hyperplasia PTLD; CHL-PTLD: classic Hodgkin lymphoma PTLD; EBV: Epstein–Barr virus; EBV-PTLD: EBV-related PTLD; EL-PTLD: early lesion PTLD.

**Table 2:** Quality investigation of the included studies.

SL	Study ID	Reference	1	2	3	4	5	6	7	8	9	Overall Score (%)
1	Dias 2018	[17]	Y	Y	Y	Y	Y	Y	Y	Y	N	88.89%
2	Fu 2017	[18]	Y	Y	Y	Y	Y	Y	Y	Y	Y	100%
3	Fujimoto 2019	[19]	Y	Y	Y	Y	Y	Y	Y	Y	Y	100%
4	Chiereghin 2016	[20]	Y	Y	Y	Y	Y	Y	N	N	Y	77.78%
5	Luo 2014	[21]	Y	Y	Y	Y	Y	Y	Y	Y	Y	100%
6	Kalra 2018	[22]	Y	Y	Y	N	Y	Y	Y	Y	N	77.78%
7	Overkamp 2020	[23]	Y	Y	Y	N	Y	Y	Y	Y	Y	88.89%
8	Yoon 2014	[24]	Y	Y	Y	Y	Y	Y	Y	Y	N	88.89%
9	Lückemeier 2023	[25]	Y	Y	Y	N	Y	Y	Y	Y	N	77.78%
10	Kinzel 2022	[26]	Y	Y	Y	Y	Y	Y	Y	Y	Y	100%
11	Sundin 2006	[27]	Y	Y	Y	Y	Y	Y	Y	Y	Y	100%
12	Fu 2016	[28]	Y	Y	Y	Y	Y	Y	Y	Y	Y	100%
13	Park 2018	[29]	Y	Y	Y	Y	Y	Y	Y	Y	Y	100%
14	Rosello 2021	[30]	Y	Y	Y	Y	Y	Y	Y	Y	Y	100%
15	Choi 2010	[31]	Y	Y	Y	Y	Y	Y	Y	Y	N	88.89%
16	Liu 2018	[32]	Y	Y	Y	Y	Y	Y	Y	Y	Y	100%
17	Pagliuca 2019	[33]	Y	Y	Y	Y	Y	Y	Y	Y	Y	100%
18	Watson 2020	[34]	Y	Y	Y	Y	Y	Y	Y	Y	Y	100%
19	Chen 2012	[35]	Y	Y	Y	N	Y	Y	N	N	Y	66.67%
20	Gunduz 2017	[36]	Y	Y	Y	Y	Y	Y	Y	Y	Y	100%
21	Fujimoto 2020	[37]	Y	Y	Y	Y	Y	Y	Y	Y	Y	100%
22	Czyzewski 2012	[38]	Y	Y	Y	N	Y	Y	N	N	N	55.55%
23	Ali 2018	[39]	Y	Y	Y	Y	Y	Y	Y	Y	N	88.89%
24	Wareham 2018	[40]	Y	Y	Y	Y	Y	Y	Y	Y	N	88.89%
25	Courville 2016	[41]	Y	Y	Y	Y	Y	Y	Y	Y	N	88.89%
26	Hahn 2023	[42]	Y	Y	Y	Y	Y	Y	Y	Y	N	88.89%
27	Styczynski 2013	[43]	Y	Y	Y	Y	Y	Y	Y	Y	Y	100%
28	Xu 2015	[44]	Y	Y	Y	Y	Y	Y	Y	Y	N	88.89%
29	Gilman 2017	[45]	Y	Y	Y	Y	Y	Y	Y	Y	Y	100%
30	Gonzalez-Farre 2014	[46]	Y	Y	Y	Y	Y	Y	Y	Y	N	88.89%
31	Kroll 2011	[47]	Y	Y	Y	Y	Y	Y	Y	Y	N	88.89%
32	Zhou 2022	[48]	Y	Y	Y	Y	Y	Y	Y	Y	Y	100%
33	D’Aveni 2014	[49]	Y	Y	Y	Y	Y	Y	N	N	N	66.67%
34	A Nash 2003	[50]	Y	Y	Y	Y	Y	Y	Y	Y	Y	100%
35	Comoli 2007	[51]	Y	Y	Y	Y	Y	Y	Y	Y	N	88.89%
36	Blaes 2009	[52]	Y	Y	Y	Y	Y	Y	Y	Y	N	88.89%
37	Comoli 2007	[53]	Y	Y	Y	Y	Y	Y	Y	Y	N	88.89%
38	Park 2006	[54]	Y	Y	Y	Y	Y	Y	Y	Y	N	88.89%
39	Althubaiti 2019	[55]	Y	Y	Y	N	Y	Y	Y	Y	Y	88.89%

1. Was the research question appropriate? 2. Is the target/study population clearly defined? 3. Were any inclusion and/or exclusion criteria mentioned? 4. Was any timeframe mentioned? 5. Are non-responders clearly described? 6. Does the sample represent the target population? 7. Were data collection methods standardized? 8. Was the timeframe sufficient so that one could reasonably expect to see an association between exposure and outcome if it existed? 9. Did the authors use statistical analyses? Y: yes; N: no. Here, <50% indicated low quality and high bias risk, 50–70% indicated moderate quality, 70–80% indicated moderately high quality, and >80% indicated high quality and low bias risk.

## Data Availability

This article is based exclusively on data obtained from previously published literature. No new datasets were generated or analyzed during this study. All data are available in the manuscript; therefore, data sharing is not applicable.

## Supplementary Materials

The following supporting information can be downloaded at: https://www.mdpi.com/article/10.3390/jcm15103867/s1. File S1. PRISMA 2020 checklist.
